# Interstitial fluid *N*-glycans serve as a proxy for serum biomarker discovery in a pilot study

**DOI:** 10.1038/s41598-026-51768-5

**Published:** 2026-07-17

**Authors:** Radka Saldova, Talus K. Gaymore, Caitriona Walsh, Eder Zavala, Paula M. Mendes

**Affiliations:** 1 National Institute for Bioprocesing Research and Training (NIBRT), Dublin, Ireland; 2 UCD School of Medicine, College of Health and Agricultural Science (CHAS), University College Dublin (UCD), Dublin, Ireland; 3 Science Foundation Ireland Research Centre for Medical Devices, Biomedical Sciences, CÚRAM, University of Galway, Galway, Ireland; 4 Centre for Biological Timing, Division of Diabetes, Endocrinology & Gastroenterology, School of Medical Sciences, Faculty of Biology, Medicine and Health, University of Manchester, Manchester, UK; 5 School of Chemical Engineering, University of Birmingham, Birmingham, UK; 6 Department of Engineering, Kanbar College of Design, Engineering and Commerce, Thomas Jefferson University, Philadelphia, PA USA

**Keywords:** Interstitial fluid, Plasma, *N*-glycans, Non-invasive, Biomarker, Sensor, Biochemistry, Biological techniques, Biomarkers

## Abstract

Protein glycosylation is the most prevalent post-translational modification and is known to undergo profound alterations in disease. While serum *N*-glycosylation has been extensively studied, interstitial fluid is gaining prominence as a minimally invasive biomarker source, underscored by the widespread clinical adoption of wearable glucose monitors in diabetic patients. However, the glycomic landscape of interstitial fluid remains largely unexplored. Here, we have performed a comparative analysis of the *N*-glycome from interstitial fluid and matched plasma from five healthy volunteers using liquid chromatography, mass spectrometry and exoglycosidase digestions. Strikingly, the interstitial fluid *N*-glycome closely shows overlapping profiles with the plasma of each individual, with only subtle differences that did not reach statistical significance. Glycosylation profiles were highly individualised, reinforcing the biological specificity of the glycome of each person. These findings demonstrate that interstitial fluid closely reflects systemic *N*-glycosylation and therefore may represent a powerful, accessible matrix for glycan biomarker discovery. They further support the development of minimally invasive or wearable technologies for monitoring glycosylation-based indicators of disease onset, progression and treatment response.

## Introduction

Glycosylation is the enzymatic process whereby a glycan group is attached to a functional group of another biomolecule. It is the most common post-translational modification of macromolecules in eukaryotes, influencing intracellular interactions, protein folding and stability, as well as receptor binding and recognition^1^. Glycosylation depends on genetic and environmental factors and is altered in many diseases including chronic inflammatory conditions and cancer^2^. *N*-glycans are one of the common classes of glycans found on proteins^2^. The main sources of serum glycoproteins are the liver and immunoglobulin-secreting plasma cells, making the total serum *N*-glycome a useful tool for detecting physiological alterations^2^. Changes in serum *N*-glycans are common in acute and chronic inflammation, cancer and other diseases, positioning *N*-glycans as promising biomarkers for diagnosing and monitoring disease progression^2^.

Biomarker analytes are most commonly measured in blood, urine, and saliva^3^. Although blood is rich in biomarkers and readily accessible throughout the body, its collection at the volumes required for diagnostics is invasive and requires trained personnel. Urine and saliva are easier to collect but contain fewer biomarkers, limiting their diagnostic utility^3^. Biomarker detection through sweat is also challenging because sweat composition fluctuates with pH, temperature, flow rate, and salinity^4,5^. Together, these limitations have increased interest in interstitial fluid (ISF). ISF has garnered attention due to its high abundance in biomarkers, the availability of analysis methods, and its nonclotting ability^6^.

ISF constitutes 75% of the body’s extracellular fluid and between 15 and 25% of an individual total body weight, with a volume thrice that of blood^4,6^. It serves as a systemic delivery system through which cells receive nutrients and oxygen, exchange inter-cellular signals and eliminate waste^4^. ISF is easily accessible, its extraction is minimally invasive and has a biomarker composition relatively equivalent to that found in the blood^4^. Approximately 92% of RNA species, 90% of circulating proteins, and 99% of proteins identified in the blood are present in ISF^4^, while some smaller proteins unique to ISF are not detectable in blood^7^. The volume of ISF in the dermis varies with skin depth, with the middle dermis containing the highest ISF content and offering optimal access due to its proximity to capillaries^4^. ISF has already demonstrated value in several clinical and research contexts. High-resolution endocrine profiling studies have shown that continuous sampling of ISF can capture detailed 24-hour hormone dynamics in humans, confirming that ISF reflects systemic physiology and can be collected with minimal disruption to normal activity^8^. In the brain, ISF can be monitored *in vivo* using microelectrode biosensors, providing high-resolution measurements of metabolic and neurochemical dynamics in response to physiological or pathological stimuli^9^. In oncology, ISF has been linked to tumor biology and clinical outcomes in breast cancer patients, suggesting that profiling *N*-glycans from proximal breast tumor fluids may help identify tumor-derived glyco-signature(s) in blood^10^.

More recent work has shown that protein biomarkers can also be detected in dermal ISF using microneedle patches equipped with integrated immunoassays and ultrabright fluorescent labels, enabling ultrasensitive quantification of proteins that are difficult to measure reliably in blood^11^. These advances highlight the increasing feasibility of macromolecular analysis in ISF, yet comprehensive characterisation of larger biomolecules remains limited. Despite this progress, ISF is still predominantly explored for metabolite sensing, and its macromolecular composition remains poorly defined, particularly with respect to protein glycosylation. The *N*-glycan profile of dermal ISF has not been characterised, and it is unclear whether ISF reflects systemic *N*-glycosylation as measured in plasma. Clarifying this relationship is essential for assessing ISF as a minimally invasive matrix for glycan biomarkers. Here, we compare *N*-glycans in dermal ISF and matched plasma to evaluate the extent to which ISF captures systemic glycosylation features.

## Results and discussion

ISF and plasma were collected from five healthy volunteers. Protein concentrations in ISF were approximately threefold lower than in plasma (Table 1), consistent with the fact that the total volume of ISF in the human body is about three times greater than that of blood^4^. For this reason, a threefold larger ISF volume was used for *N*-glycan analysis to obtain comparable results. *N*-glycans were released from all samples and profiled on Hydrophilic Interaction Liquid Chromatography - Ultra Performance Liquid Chromatography (HILIC-UPLC) using fluorescent labelling with 2-aminobenzamide (2AB) enabling reliable quantitation across analytical runs^12^. Liquid chromatography - mass spectrometry (LC-MS) combined with HILIC-UPLC and exoglycosidase digestions were performed for detailed structural analyses of ISF and plasma pools.

**Table 1:** Protein concentration in all samples.

Sample ID	Protein concentration (mg/ml)	
	ISF	plasma
subject 1	24.20	78.90
subject 2	28.10	76.50
subject 3	38.00	94.10
subject 4	33.40	95.60
subject 5	30.90	81.00

### ISF and plasma pools contain the same set of *N*-glycans

**Fig. 1:**
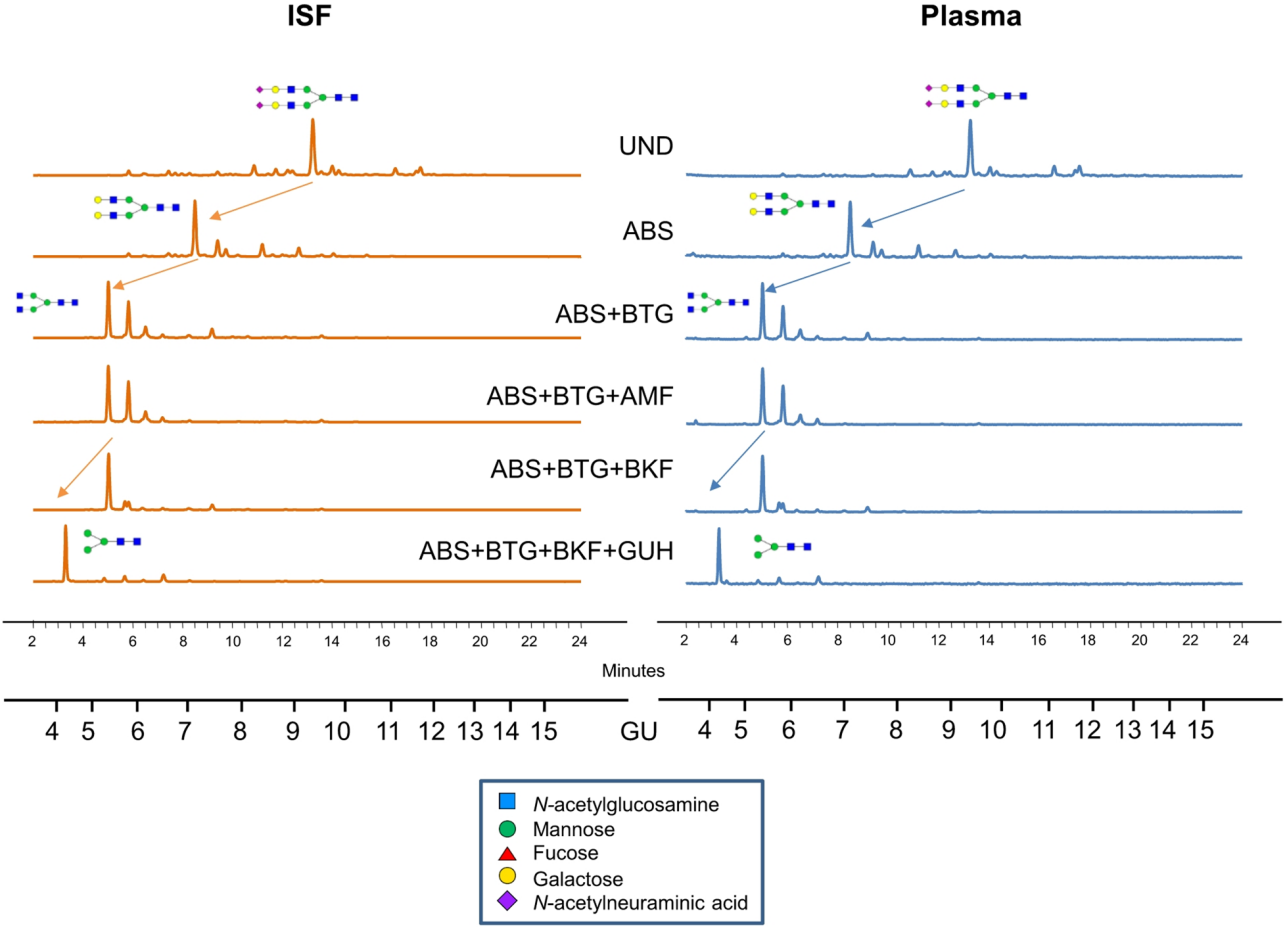
HILIC-UPLC chromatograms of ISF and plasma from pooled ISF and plasma undigested (UND) and digested with sequential arrays of exoglycosidase enzymes, incorporating *Arthrobacter ureafaciens *sialidase (ABS), bovine testes β-galactosidase (BTG), almond meal α-fucosidase (AMF), bovine kidney α-fucosidase (BKF),β-*N*-acetylglucosaminidase cloned from *S. pneumoniae*, expressed in *Escherichia coli * (GUH). Main glycan (A2G2S2 is traced through the digests).

For detailed glycan characterization, equal amounts from each ISF sample were pooled, and equal amounts from each plasma sample were combined, ensuring sufficient material and equal representation of all samples for comprehensive analysis. These pooled samples were analyzed using LC-MS (Table S1) and HILIC-UPLC combined with exoglycosidase digestions (Fig. 1).

The LC-MS analyses focused on masses of *N*-glycan structures typically found in serum or plasma, as previously described^12–16^. Although serum and plasma share the same repertoire of *N*-glycans, they can differ slightly in the relative abundance of specific structures, such as biantennary digalactosylated monosialylated (A2G2S1) and biantennary digalactosylated disialylated (A2G2S2) glycans. These differences are largely attributed to the presence of fibrinogen in plasma, a protein absent in serum^18^. Despite these nuances, both ISF and plasma samples displayed the same set of structures of *N*-glycans as listed in Table 2 and Table S1.

**Table 2:** List of glycans found in ISF and plasma pool from the same individuals.

*GP	ISF		Plasma		Glycan Structure	Monosaccharide composition
	GU	% Area	GU	% Area		
**1**	4.82	0.04	4.70	0.52	**A1**	HexNAc_3_Hex_3_
**2**	5.38	0.12	5.41	1.08	**M4**	Hex_4_
					**FA1**	dHex_1_HexNAc_3_Hex_3_
					**A2**	HexNAc_4_Hex_3_
**3**	5.49	0.01	5.51	0.18	**A1G1**	HexNAc_3_Hex_4_
**4**	5.66	0.03	5.68	0.23	**A2B**	HexNAc_5_Hex_3_
					**A1G1**	HexNAc_3_Hex_4_
**5**	5.84	2.30	5.84	1.71	**FA2**	dHex_1_HexNAc_4_Hex_3_
**6**	6.16	1.49	6.16	1.51	**M5**	Hex_5_
					**FA2B**	dHex_1_HexNAc_5_Hex_3_
					**FA1G1**	dHex_1_HexNAc_3_Hex_4_
					**A2G1**	HexNAc_4_Hex_4_
					**A2G1**	HexNAc_4_Hex_4_
**7**	6.52	0.20	6.50	0.41	**A2BG1**	HexNAc_5_Hex_4_
**8**	6.65	2.24	6.66	1.36	**FA2G1**	dHex_1_HexNAc_4_Hex_4_
					**A2BG1**	HexNAc_5_Hex_4_
					**M4A1G1**	HexNAc_3_Hex_5_
**9**	6.78	1.20	6.78	0.85	**FA2G1**	dHex_1_HexNAc_4_Hex_4_
**10**	6.90	0.98	6.89	0.75	**FA2BG1**	dHex_1_HexNAc_5_Hex_4_
**11**	7.05	1.23	7.05	0.99	**FA2BG1**	dHex_1_HexNAc_5_Hex_4_
					**M6**	Hex_6_
**12**	7.15	0.27	7.14	0.72	**A1G1S1**	HexNAc_3_Neu5Ac_1_Hex_4_
					**A2G2**	HexNAc_4_Hex_5_
**13**	7.33	0.20	7.29	0.33	**A1G1S1**	HexNAc_3_Neu5Ac_1_Hex_4_
					**A2BG2**	HexNAc_5_Hex_5_
**14**	7.56	2.36	7.55	1.59	**M5A1G1**	HexNAc_3_Hex_6_
					**FA2G2**	dHex_1_HexNAc_4_Hex_5_
					**A2G1S1**	HexNAc_4_Neu5Ac_2_Hex_4_
					**A2G1S1**	HexNAc_4_Neu5Ac_2_Hex_4_
					**FA1G1S1**	dHex_1_HexNAc_3_Neu5Ac_2_Hex_4_
**15**	7.70	0.58	7.71	0.53	**FA2BG2**	dHex_1_HexNAc_5_Hex_5_
					**M7**	Hex_7_
					**A2BG1S1**	HexNAc_5_Neu5Ac_1_Hex_4_
**16**	7.78	0.84	7.77	0.75	**A2BG1S1**	HexNAc_5_Neu5Ac_1_Hex_4_
					**FA2G1S1**	dHex_1_HexNAc_4_Neu5Ac_1_Hex_4_
					**M4A1G1S1**	HexNAc_3_Neu5Ac_1_Hex_5_
					**M7**	Hex_7_
**17**	7.88	0.66	7.87	0.52	**FA2G1S1**	dHex_1_HexNAc_4_Neu5Ac_1_Hex_4_
					**FA2BG1S1**	dHex_1_HexNAc_5_Neu5Ac_1_Hex_4_
**18**	7.94	0.38	7.97	0.53	**FA2BG1S1**	dHex_1_HexNAc_5_Neu5Ac_1_Hex_4_
**19**	8.22	6.00	8.22	3.85	**A2G2S1**	HexNAc_4_Neu5Ac_1_Hex_5_
					**A3G3**	HexNAc_5_Hex_6_
**20**	8.36	0.28	8.35	0.42	**A2BG2S1**	HexNAc_5_Neu5Ac_1_Hex_5_
**21**	8.48	1.01	8.48	0.96	**M5A1G1S1**	HexNAc_3_Neu5Ac_1_Hex_6_
					**FA3G3**	dHex_1_HexNAc_5_Hex_6_
					**M8**	Hex_8_
					**A2G2S1**	HexNAc_4_Neu5Ac_1_Hex_5_
**22**	8.62	3.69	8.62	2.53	**FA2G2S1**	dHex_1_HexNAc_4_Neu5Ac_1_Hex_5_
					**M8**	Hex_8_
**23**	8.85	3.68	8.85	2.91	**FA2BG2S1**	dHex_1_HexNAc_5_Neu5Ac_1_Hex_5_
**24**	8.94	3.31	8.95	2.89	**A2F1G2S1**	dHex_1_HexNAc_4_Neu5Ac_1_Hex_5_
					**A2G2S2**	HexNAc_4_Neu5Ac_2_Hex_5_
					**FA2G2S2**	dHex_1_HexNAc_4_Neu5Ac_2_Hex_5_
**25**	9.35	32.71	9.35	26.99	**A2G2S2**	HexNAc_4_Neu5Ac_2_Hex_5_
					**M9**	Hex_9_
					**A3G3S1**	HexNAc_5_Neu5Ac_1_Hex_6_
**26**	9.52	3.23	9.52	2.71	**FA3G3S1**	dHex_1_HexNAc_5_Neu5Ac_1_Hex_6_
					**A2BG2S2**	HexNAc_5_Neu5Ac_2_Hex_5_
**27**	9.75	5.92	9.75	5.04	**A3F1G3S1**	dHex_1_HexNAc_5_Neu5Ac_1_Hex_6_
					**FA2G2S2**	dHex_1_HexNAc_4_Neu5Ac_2_Hex_5_
**28**	9.88	3.01	9.89	2.99	**FA2BG2S2**	dHex_1_HexNAc_5_Neu5Ac_2_Hex_5_
					**A2F1G2S2**	dHex_1_HexNAc_4_Neu5Ac_2_Hex_5_
					**M9Glc**	HexNAc_1_Hex_9_
**29**	10.02	1.29	10.02	1.39	**A3G3S2**	HexNAc_5_Neu5Ac_2_Hex_6_
**30**	10.27	0.51	10.30	0.74	**-**	-
**31**	10.47	0.79	10.47	0.83	**FA3G3S2**	dHex_1_HexNAc_5_Neu5Ac_2_Hex_6_
					**A3G3S2**	HexNAc_5_Neu5Ac_2_Hex_6_
**32**	10.72	1.17	10.74	1.72	**A3G3S3**	HexNAc_5_Neu5Ac_3_Hex_6_
					**A3F1G3S2**	dHex_1_HexNAc_5_Neu5Ac_2_Hex_6_
**33**	10.85	0.41	10.89	0.60	**A3G3S3**	HexNAc_5_Neu5Ac_3_Hex_6_
**34**	11.13	4.57	11.14	5.84	**FA3G3S3**	dHex_1_HexNAc_5_Neu5Ac_3_Hex_6_
					**FA3BG3S3**	dHex_1_HexNAc_6_Neu5Ac_3_Hex_6_
					**A4G4S2**	HexNAc_6_Neu5Ac_2_Hex_7_
					**A3G3S3**	HexNAc_5_Neu5Ac_3_Hex_6_
**35**	11.27	0.71	11.28	0.83	**-**	-
**36**	11.46	0.69	11.47	0.82	**FA3G3S3**	dHex_1_HexNAc_5_Neu5Ac_3_Hex_6_
**37**	11.61	2.33	11.62	2.82	**A3G3S3**	HexNAc_5_Neu5Ac_3_Hex_6_
					**A3F1G3S3**	dHex_1_HexNAc_5_Neu5Ac_3_Hex_6_
					**FA3BG3S3**	dHex_1_HexNAc_6_Neu5Ac_3_Hex_6_
**38**	11.71	4.66	11.71	5.45	**A4G4S3**	HexNAc_6_Neu5Ac_3_Hex_7_
					**A3F1G3S3**	dHex_1_HexNAc_5_Neu5Ac_3_Hex_6_
**39**	11.91	0.87	11.85	1.44	**A4G4S3**	HexNAc_6_Neu5Ac_3_Hex_7_
**40**	12.02	0.75	12.03	1.20	**A4F1G3S3**	dHex_1_HexNAc_6_Neu5Ac_3_Hex_6_
					**A3F2G3S3**	dHex_2_HexNAc_5_Neu5Ac_3_Hex_6_
**41**	12.45	0.87	12.47	2.09	**A4G4S4**	HexNAc_6_Neu5Ac_4_Hex_7_
					**A3F2G3S3**	dHex_2_HexNAc_5_Neu5Ac_3_Hex_6_
**42**	12.77	1.04	12.78	2.49	**A4G4S4**	HexNAc_6_Neu5Ac_4_Hex_7_
**43**	13.28	0.61	13.29	1.46	**A4G4S4**	HexNAc_6_Neu5Ac_4_Hex_7_
**44**	13.61	0.37	13.64	1.12	**A4G4S4**	HexNAc_6_Neu5Ac_4_Hex_7_
					**FA4G4S4**	dHex_1_HexNAc_6_Neu5Ac_4_Hex_7_
**45**	13.84	0.23	13.86	1.33	**A4F1G4S4**	dHex_1_HexNAc_6_Neu5Ac_4_Hex_7_
**46**	14.31	0.19	14.62	1.95	**A4F2G4S4**	dHex_2_HexNAc_6_Neu5Ac_4_Hex_7_
					**A4F3G4S4**	dHex_3_HexNAc_6_Neu5Ac_4_Hex_7_
					**A4G4Lac1S4**	HexNAc_7_Neu5Ac_4_Hex_8_

*Same GP numbers as previously published^12^.

**Structure abbreviations**: all *N*-glycans have two core GlcNAcs; F at the start of the abbreviation indicates a core fucose *α*1,6-linked to the inner GlcNAc; Mx, number (x) of mannose on core GlcNAcs; Ax, number of antenna (GlcNAc) on trimannosyl core; A2, biantennary with both GlcNAcs as *β*1,2-linked; A3, triantennary with a GlcNAc linked *β*1,2 to both mannose and the third GlcNAc linked *β*1,4 to the *α*1,3 linked mannose; A4, GlcNAcs linked as A3 with additional GlcNAc *β*1,6 linked to *α*1,6 mannose; B, bisecting GlcNAc linked *β*1,4 to *β*1,3 mannose; Gx, number (x) of *β*1,4 linked galactose on antenna; F(x), number (x) of fucose linked *α*1,3 to antenna GlcNAc; Sx, number (x) of sialic acids linked to galactose. **Monosaccharide abbreviations**: Hex=hexose, HexNAc=*N*-acetylhexosamine, dHex=deoxyhexose, Neu5Ac=*N*-acetylneuraminic acid.

Importantly, the pooled HILIC-UPLC chromatograms and the additional exoglycosidase digestion profiles also revealed a similarly rich repertoire of glycans in ISF and plasma, with no major qualitative differences between the two fluids and consistent with previously published serum *N*-glycome profiles^12^ (Fig. 1). Exoglycosidase enzymes digest specific *N*-glycan residues and the resulting shifts in glucose units (GU) values were used to assign individual glycan structures (Fig. 1)^18^. The major structure A2G2S2 was digested to the core mannose M3 with the combined enzymes together, *Arthrobacter ureafaciens *sialidase (ABS) digested sialic acids, bovine testes β-galactosidase (BTG) galactoses and GUH GlcNAc residues (Fig. 1)^12,18^.

**Fig. 2:**
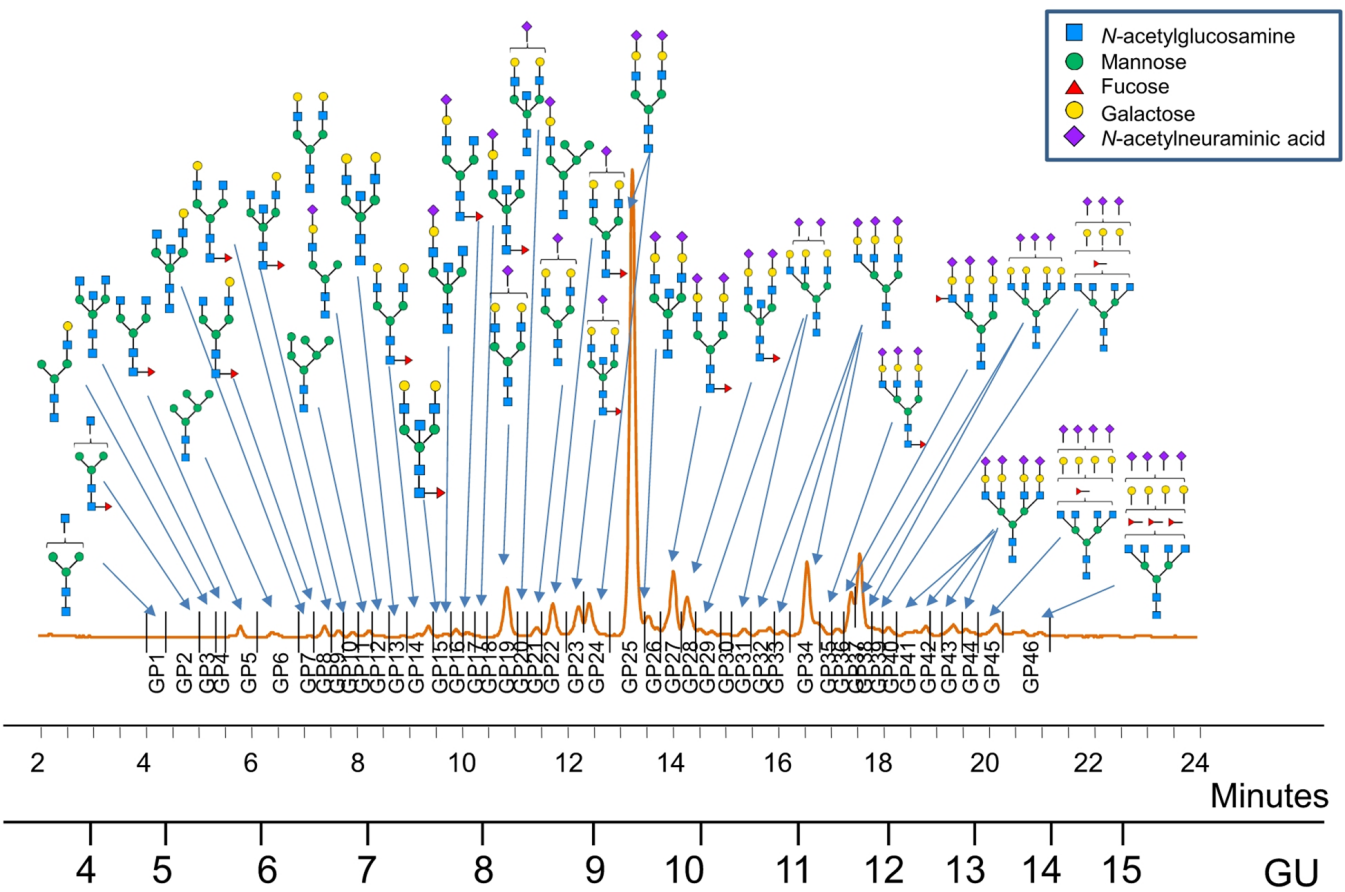
HILIC-UPLC chromatogram of pooled ISF and separation into 46 GPs.

Resulting from LC-MS analyses combined with exoglycosidase digestions, both plasma and ISF pools contained all major classes of *N*-glycans, including complex, hybrid and oligomannosylated structures as indicated by shifts in GUs by enzymes digesting sialic acids, galactoses, fucoses and GlcNAc residues (Fig. 2; Table 2, Table S1). These included glycans with and without core fucose, up to three outer-arm fucoses and antennary structures ranging from mono- to tetraantennary. The number of galactose residues ranged from none to four, with biantennary digalactosylated disialylated glycans (A2G2S2, GP25) being the most abundant, reflecting patterns established in earlier glycomic analyses^12^ (Fig. 2; Table 2, Table S1). These abundant glycan structures are primarily derived from acute-phase proteins and immunoglobulins^12,13,15^ suggesting that the ISF contains the same dominant glycoprotein contributors as plasma^4^.

This structural similarity supports the concept that ISF can serve as a proxy for systemic glycosylation, given that the same hallmark glycan features observed in blood are also detectable in the interstitial compartment. This observation is consistent with the physiological origin of ISF as a filtrate of plasma. ISF is formed by capillary filtration and contains a broad range of circulating proteins, with composition largely overlapping that of plasma, particularly within the molecular weight range of most glycoproteins^19^^,20^. Proteomic studies have demonstrated a strong overlap between ISF and plasma protein composition, indicating that ISF reflects systemic molecular signatures^7^^,21^. As *N*-glycans are covalently linked to proteins and are not modified during passive transport processes, their structural integrity is preserved during exchange between plasma and the interstitial compartment.

Importantly, the glycan assignments were cross-validated using combined HILIC-UPLC with fluorescence detection (FLD) and electrospray ionization mass spectrometry (ESI-MS) workflow, which provided both relative quantitation and detailed structural composition for each glycan peak^12^^,22^. The consistency between enzymatic digestion patterns and MS-based identifications enhances confidence in the accuracy of glycan characterization. No novel or unexpected glycan structures were found unique to ISF. Instead, ISF contained a representative subset of the circulating *N*-glycome. This finding indicates that the major *N*-glycosylation motifs accessible through blood sampling are also present in dermal ISF, which is a critical prerequisite for establishing ISF as a surrogate biofluid for glycomic biomarker discovery.

### Reproducibility and inter-individual consistency in glycan profiling

**Fig. 3:**
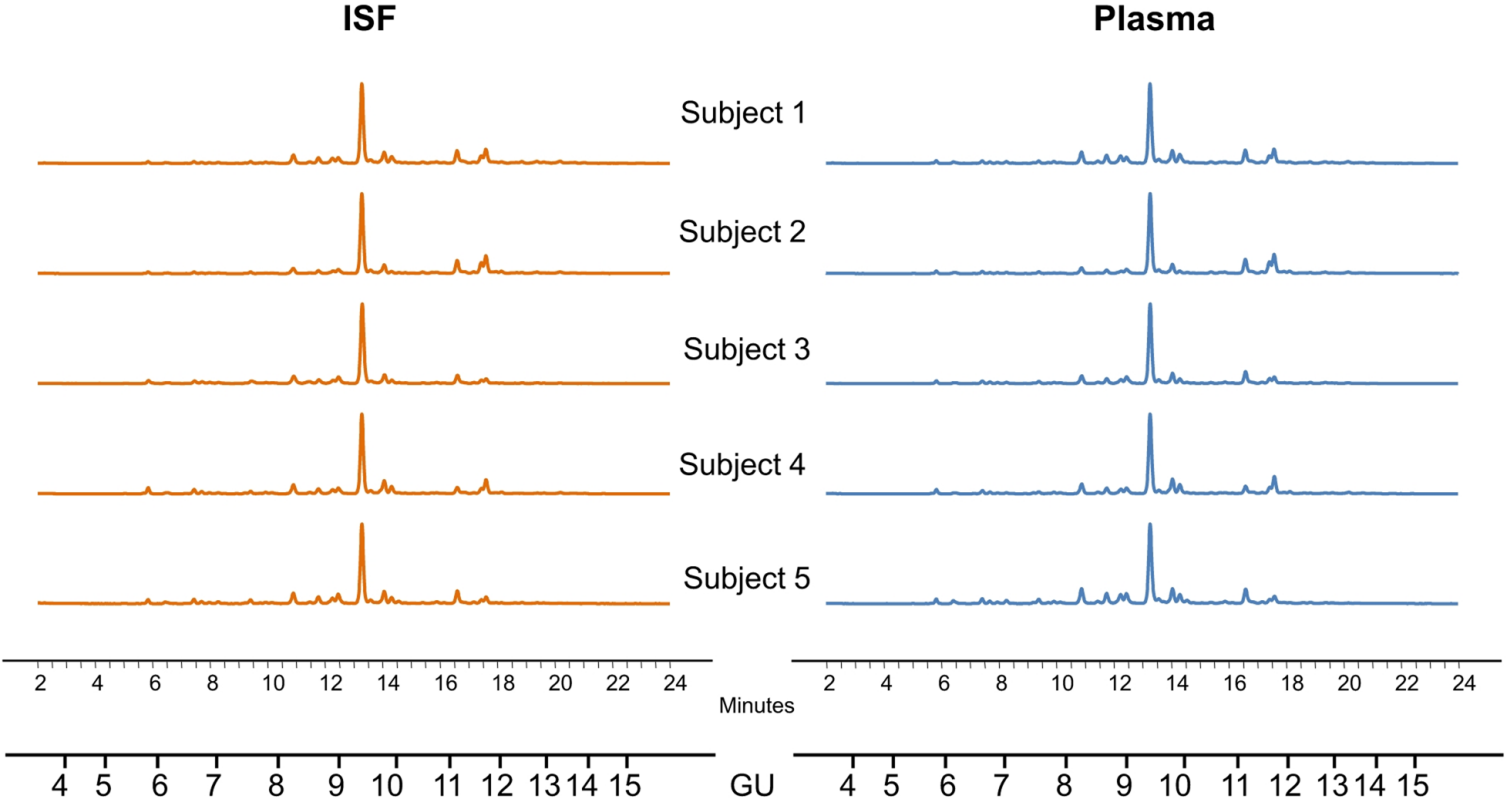
HILIC-UPLC chromatograms of ISF and plasma from 5 individuals.

Following the pooled sample analyses used for structural glycan characterization, individual ISF and plasma samples were profiled using HILIC-UPLC to assess inter-individual variability and evaluate the reproducibility of the analytical workflow. Each resulting HILIC-UPLC chromatogram was separated into 46 glycan peaks (GPs) (Fig. 2) and derived features were calculated as previously published^12^. While pooled analyses provided a comprehensive overview of glycan structures, individual profiling enabled assessment of biological variability and allowed for statistical and multivariate comparisons across matched subjects. The glycan profiles from individual ISF samples closely resembled those from matched plasma samples, consistent with the patterns observed in the pooled data (Fig. 2 and Fig. 3). This supports the reliability of the HILIC-UPLC platform for glycan profiling. Technical replicates showed consistent glycan peak patterns and relative abundances (Table S2). While variability was observed for some lower-abundance glycans, as reflected in the coefficients of variation, the overall profiles remained consistent across samples, in line with the established high precision of HILIC-UPLC in glycomics^12^.

The strong concordance between pooled and individual datasets, together with the high reproducibility of HILIC-UPLC and LC-MS, confirms that the observed glycan patterns are biologically meaningful and not an artifact of sample handling or analysis.

### Glycan profiles are individual specific and consistent across ISF and plasma

**Fig. 4:**
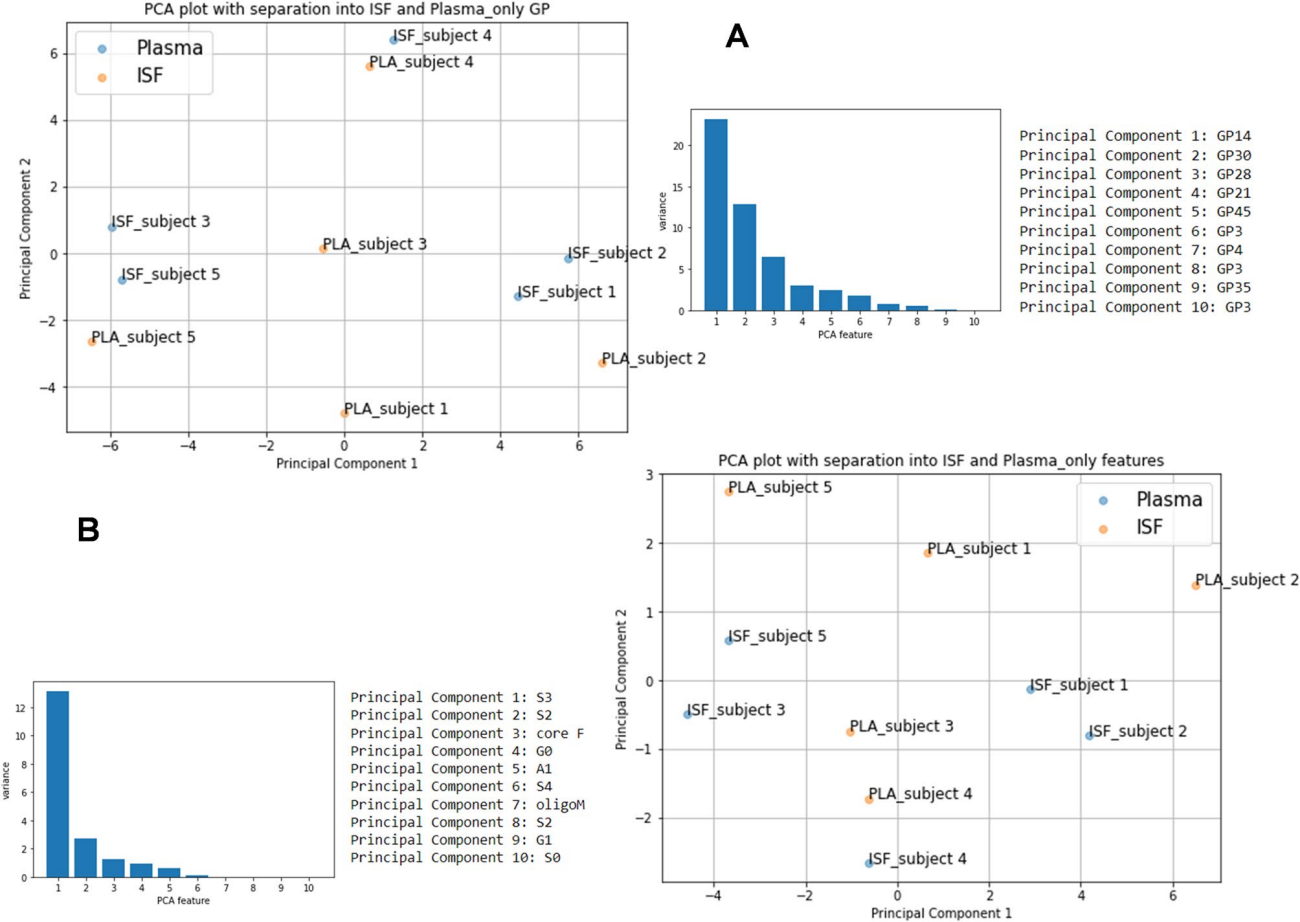
PCA plots cluster the individual subjects rather than ISF and plasma samples, based on GPs (**A**) and features (**B**) peak areas. The individual peak areas for each GP and feature in each subject are in Table S2, average areas were taken from each replicate.

One of the most striking outcomes of this study was revealed through unsupervised principal component analysis (PCA): samples clustered predominantly by sample donor identity rather than by biofluid type (Fig. 4). Ten principal components were plotted for assessments of how they contributed to the separation. The first three glycan peaks in these principal components contributing most strongly to this separation included GP14 (primarily core fucosylated biantennary digalactosylated glycans, FA2G2), GP30 (primarily tetraantennary tetragalactosylated monosialylated glycans, A4G4S1), and GP28 (mainly core fucosylated biantennary digalactosylated disialylated glycans, FA2BG2S2) along with features corresponding to trisialylated (S3), disialylated (S2) and core fucosylated (coreF) structures (Fig. 4). These results indicate that the inter-individual variation in *N*-glycan profiles is much greater than intra-individual differences between ISF and plasma from the same individual. Biologically, this suggests that each person has a characteristic glycomic profile that remains consistent across physiological compartments. Prior large-scale glycomic studies have shown that glycan profiles are individual specific and stable over time within the same individual^23^^[,24^. In fact, inter-individual glycan variability has been reported to exceed that observed in proteomic or genomic data^23^. Our findings are consistent with this concept: the ISF *N*-glycome appears to show an overlapping profile with the plasma *N*-glycome for each individual (Fig. 3), and by correlation of most GPs and features in paired fluids (Table S3). shaped by genetic and metabolic influences. The lack of separation by biofluid type in the PCA, despite physiological differences between ISF and plasma, suggests that systemic regulatory factors such as glycosyltransferase expression have a greater influence than local fluid origin in determining glycan patterns.

From a clinical perspective, this finding is highly encouraging. It suggests that minimally invasive ISF sampling can reliably capture the same individual-specific glycosylation signatures that are currently measurable only through venous blood draws. This supports the broader potential of ISF as a biofluid for glycan-based diagnostics. The observation that inter-individual variation exceeds

analytical variability confirms that the clustering by individual reflects biological differences rather than technical artifacts. These results are consistent with the concept of glyco-phenotypes, which describe stable and person-specific glycan profiles that differ across individuals^23^. The predominance of individual patterns over biofluid-specific differences (Fig. 4) indicates that disease-related glycan alterations are likely to be detectable in both ISF and plasma from the same individual. This has important implications for biomarker development and suggests that personal glycomic baselines may be necessary for accurate interpretation. Longitudinal sampling may be especially valuable for distinguishing stable individual glycan traits from changes associated with disease onset, progression, or treatment response.

## Subtle glycan differences between plasma and ISF consistent for individuals

**Fig. 5:**
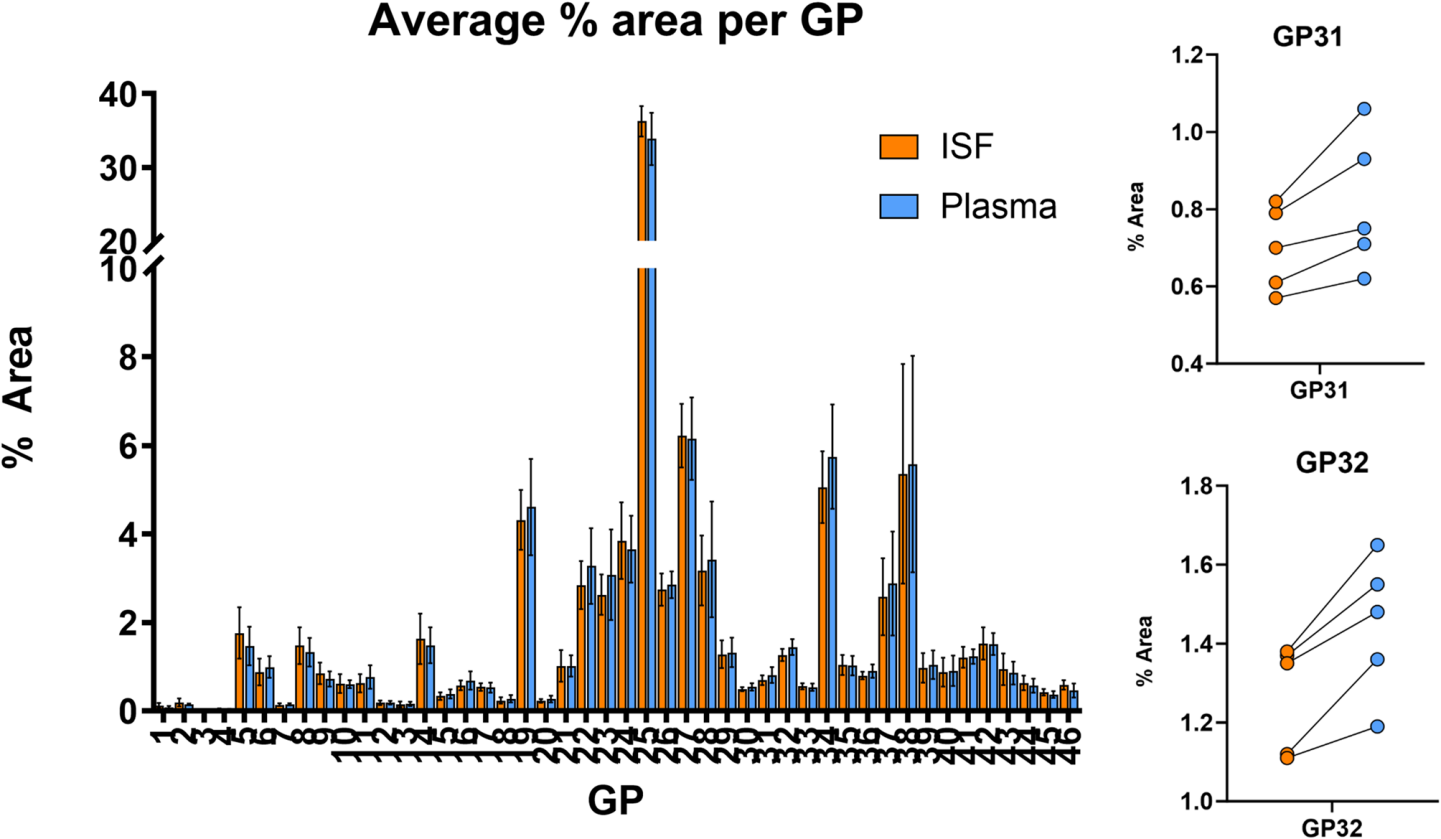
Average % of peak areas for each GP in each sample. Zoomed in are GP31 and 32 with consistent increase in the % areas in plasma. The individual peak areas for each GP plotted here are in Table S2, average areas were taken from each replicate.

Although the overall glycan profiles of ISF and plasma were remarkably similar with no statistically significant differences observed after adjustment for multiple testing (Fig. 5, Table S3), a few specific glycan peaks showed consistent differences between the two fluids. Notably, peaks GP31 and GP32 were reproducibly observed at higher relative HILIC-UPLC abundance in plasma compared to ISF for all individuals (Fig. 5, Table S2**)**, despite not reaching statistical significance in this small sample set (Table S3). These peaks represent mainly triantennary trigalactosylated di- and trisialylated glycans, namely A3G3S2 and A3G3S3 (Table 2, Table S1)^12^.

Differences were observed not only in the relative HILIC-UPLC peak areas, but also in the composition of glycan structures within these peaks, as quantitated by LC-MS. In GP31, the proportion of core fucosylated and non fucosylated triantennary trigalactosylated disialylated glycans (FA3G3S2 and A3G3S2) varied slightly between plasma and ISF. ISF samples contained a higher proportion of core fucosylated structures (FA3G3S2, 18.87% vs. 14.87%), although non fucosylated forms dominated in both fluids (A3G3S2, 81.13% vs. 85.13%). In GP32, ISF exhibited a higher proportion of outer arm fucosylated triantennary trigalactosylated disalylated glycans (A3F1G3S2 isomers, 53.38%) relative to triantennary trigalactosylated trisialylated glycans (A3G3S3, 46.62%) when compared with pooled plasma (41.28% and 58.72% respectively). These results suggest a subtle enrichment of both core and outer arm fucosylated triantennary trigalactosylated glycans in ISF compared to their non fucosylated counterparts and to corresponding structures in plasma. To our knowledge, fucosylation differences in ISF relative to plasma have not been previously reported.

Highly branched glycans such as those found in GP31 and GP32 (Table 2, Table S1) are typically carried by abundant large, and heavily glycosylated plasma acute-phase glycoproteins^12,13,15.^ One plausible explanation for their lower relative abundance in ISF is that high molecular weight glycoproteins, although abundant in plasma, may be less able to traverse the capillary endothelium due to size and charge restrictions. Smaller plasma proteins may equilibrate more readily, while larger or membrane-bound glycoproteins may enter ISF less efficiently. Alternatively, these glycoproteins may be present in ISF but at lower concentrations due to dilution or local metabolic turnover.

The consistently higher GP31 and GP32 signals observed in plasma across individuals (Fig. 5), despite not reaching statistical significance after adjustment for multiple testing *p* < 0.05 (Table S3), suggest a potential biological difference that may be confirmed in studies with larger cohorts. Interestingly, these two GPs did reach statistical significance and had lowest p-values from all GPs before adjustment for multiple testing (*p* = 0.029 for GP31 and *p* = 0.007 for GP32). Importantly, beyond these two specific peaks, no broad differences were found in glycan class distributions. Total levels of fucosylation, galactosylation and sialylation were comparable between ISF and plasma (Table S3), indicating that ISF is not lacking any major glycan category. Nevertheless, small quantitative differences in selected glycan species may reflect compartment-specific protein distribution or difference in clearance from the interstitial space. However, the underlying mechanisms cannot be conclusively determined from the current data and require further investigation. Similar subtle variations between ISF and plasma or serum have been reported in prior proteomic studies^4,7^.

Future studies incorporating targeted glycoproteomic analyses could help identify the specific carrier proteins for GP31 and GP32 glycans and clarify the mechanisms underlying their differential distribution. Current findings suggest that while ISF glycome closely resembles that of plasma, subtle differences in specific glycan abundances, such as higher levels of highly sialylated multiantennary glycans in plasma, may reflect underlying differences in protein size, filtration dynamics, or compartment-specific localization.

### Clinical and biological implications of ISF glycome as a potential biomarker proxy

Our findings demonstrate that the *N*-glycan ISF profile closely reflects that of the plasma, supporting its potential as a minimally invasive surrogate for blood-based glycomic profiling. This correspondence is particularly valuable given recent advances in microneedle patch technology, which enable ISF sampling without phlebotomy^3^. The ISF glycome contains all major classes previously implicated in disease, including sialylated, fucosylated, oligomannosylated, and multiantennary structures^2^ (Fig. 2; Table 2, Table S1). These glycan features are frequently altered in cancer, inflammatory, metabolic, infectious and metabolic diseases^2,15^, and their detection in ISF suggests strong potential for diagnostic applications. This diagnostic potential is further supported by prior studies in breast cancer patients, where tumor-associated glycan alterations in tumor ISF were also reflected in matched patient’s serum^10^.

ISF is also being leveraged clinically through wearable and minimally invasive sensors, as exemplified by the US Food and Drug Administration (FDA) approval of continuous glucose monitoring in diabetic patients^25^. Advances in microneedle technology are now extending ISF monitoring beyond glucose, with an integrated microneedle platform demonstrating continuous, real-time dual-analyte sensing in human ISF during daily activity^26^. While these developments underscore the growing analytical reach of ISF-based monitoring, most current technologies remain focused on low-molecular weight targets^3,6,7,27,28,29^.

One particularly promising implication of an accessible ISF glycome is the potential for longitudinal, personalized biomarker monitoring through minimally invasive sampling. Emerging microneedle patch technologies already enable serial ISF collection and on-device analysis of protein biomarkers^11^, suggesting that similar approaches could be adapted for glycomic profiling. Because each individual’s glycan profile is stable over time and specific to that person^23,24^, serial sampling could reveal subtle glycosylation changes associated with disease processes that might be missed by one-time cross-sectional measurements. Implementing ISF glycomics in clinical settings will require addressing challenges such as small sample volumes and analytical complexity, but ongoing advances in high sensitivity glycoanalytical techniques and microfluidic systems are helping to overcome these barriers. As these technologies mature, the ISF glycome may offer a valuable reflection of both systemic and tissue-level physiological states, creating new opportunities for real-time and individualized diagnostic strategies^4,14,15^.

This study is based on a small cohort of healthy individuals derived from a single source, and future work will expand to larger and clinically diverse populations, including cancer and chronic inflammatory diseases, to assess generalisability and clinical relevance. Notably, the consistent concordance observed across paired ISF and plasma samples within individuals supports the robustness of the present findings and suggests that they are not driven by cohort-specific effects. Longitudinal sampling will also be important to evaluate the temporal stability of the ISF *N*-glycome. In addition, ISF was collected using a single sampling approach, and studies directly comparing sample collection methodologies will be valuable to confirm their equivalence^4,30^.

In conclusion, this study demonstrates that human dermal ISF contains an *N*-glycome that closely resembles that of plasma. Through structural characterization and individual-level comparisons, we show that ISF includes the same major *N*-glycan features found in blood and captures person-specific glycosylation patterns. These results support the feasibility of using ISF for glycomic profiling and lay the groundwork for its potential role in minimally invasive biomarker research. Further studies involving larger cohorts and disease contexts, as well continued improvements in ISF sampling technologies, will be important to establish clinical utility. By advancing our understanding of glycosylation in dermal fluid, this work contributes to the broader effort to expand diagnostic options beyond traditional blood draws and move toward more accessible monitoring approaches.

## Methods

### Subjects

Human dermal ISF and matched plasma samples were provided by KIFFIK BioPharma (Providence, Rhode Island, USA). The samples originated from adult Caucasian donors aged approximately 40–60 years, collected in the United States under KIFFIK’s BioPharma’s institutional review and donor consent protocols. All samples were collected in accordance with relevant guidelines and regulations, and informed consent was obtained from all participants prior to sample collection. The sample collection was conducted for internal device testing purposes and was deemed not to require institutional review board approval by KIFFIK BioPharma in accordance with applicable local regulations. All procedures were conducted in accordance with the principles of the Declaration of Helsinki.

### Interstitial fluid (ISF) collection using the KIFFIK K-Exp device

ISF samples were collected using the KIFFIK ISF collection device (KIFFIK Biomedical Inc Providence, Rhode Island, USA), a non-invasive, wearable system that continuously extracts ISF through intact skin using mild electrical and negative pressure. The device employs non-invasive electrodes to deliver micro currents (electroporation) to open up micro channels in the skin, and sealed micro cartridge, enabling capture of clear ISF in volumes sufficient for laboratory analysis. By avoiding microneedles or blistering, no contamination from sweat and epidermal lipids has been observed. Collection sessions typically lasted around 20–60 min, yielding approximately 20–50 µL of dermal ISF per donor.

Upon collection, ISF samples were aliquoted, immediately frozen on dry ice, and stored at −80 °C until analysis.

### Plasma collection

Blood was drawn by venipuncture into EDTA-coated vacutainer tubes. Samples were centrifuged at 2,000 × g for 10 min at 4 °C to separate plasma, which was carefully aliquoted into low-binding microtubes to prevent protein adsorption. Plasma aliquots were immediately frozen on dry ice and stored at − 80 °C.

### Glycan analysis

*N*-glycans were released in duplicates from the ISF and plasma samples using in-gel-block method as previously described^31^. Briefly, the samples were set into gel blocks, reduced, alkylated and washed. *N*-glycans were released by adding Peptide *N*-glycosidaseF (PNGase F) (500,000 units/mL, New England Biolabs, P0709L) as previously described^32^. The glycans were fluorescently labelled with 2AB^33,34^ and excess 2AB reagent was removed on Whatman 3MM paper in acetonitrile^22,31^.

### Hydrophilic interaction liquid chromatography-ultra performance liquid chromatography (HILIC-UPLC)

HILIC-UPLC was carried out using a BEH Glycan 1.7 μm particles in 2.1 × 150 mm column (Waters, Milford, MA) on an Acquity UPLC (Waters) equipped with a Waters temperature control module and a Waters Aquity fluorescence detector. Solvent A was 50 mM ammonium formate, pH 4.4. Solvent B was acetonitrile. The column temperature was 40 °C. The 30 min method was used with a linear gradient of 30 − 47% with buffer A at 0.56 mL/min flow rate for 23 min followed by 47 − 70% A and to complete the run, returning to 30% A^12^. The samples were injected in 70% acetonitrile and fluorescence was measured at 420 nm with excitation at 330 nm. For accurate quantification, the system was calibrated using an external standard composed of hydrolysed and 2AB-labeled glucose oligomers, which was used to create a dextran ladder with glucose units (GUs)^31^.

### UPLC-mass spectrometry (UPLC-MS)

Samples were analyzed using LC-MS on a Thermo Scientific Q Exactive Plus system. Chromatographic separation used a Waters BEH Glycan column (1.7 μm, 1.0 × 150 mm) at 60 °C. Mobile phases were 50 mM ammonium formate (pH 4.4) (Solvent A) and acetonitrile (Solvent B). A 40-minute gradient (0.15 mL/min) ran from 28% to 45% A. Samples (10 µL) were injected in 75% acetonitrile and kept at 5 °C. Fluorescence detection was set at an excitation wavelength of 320 nm and an emission wavelength of 420 nm. Mass spectrometric detection was carried out in negative mode with a spray voltage of 3.40 kV, capillary temperature of 320 °C, auxiliary gas heater temperature of 300 °C, and sheath and sweep gas flow rates of 30 and 10 L/h, respectively. MS parameters included negative mode, 3.40 kV spray voltage, 320 °C capillary temperature, 450–2500 m/z scan range, 70,000 resolution.

### Exoglycosidase digestions

The 2-AB labelled oligosaccharides were digested in volume of 10 µL for 16 h at 37 °C in 10X buffer. All enzymes were purchased from Agilent or New England Biolabs (NEB). The 2AB-labeled glycans were digested in 10 µL total volume for 18 h at 37 °C in 50 mM sodium acetate buffer, pH 5.5, using arrays of the following enzymes: *Arthrobacter ureafaciens* sialidase (ABS, removes nonreducing terminal *N*-acetylneuraminic acid (sialic acid) residues via α(2–3), α(2–6) and α(2–8) linkages, EC 3.2.1.18, NEB), 1000 U/mL; bovine testes β-galactosidase (BTG, cleaves nonreducing terminal galactose residues with β(1–3) and β(1–4) linkages, EC 3.2.1.23, Agilent), 1 U/mL; bovine kidney α-fucosidase (BKF, cleaves nonreducing terminal fucose residues linked via α(1–2) and α(1–6) rather than α(1–3) and α(1–4)-linked fucose, and also digests core α(1–6) fucose, EC 3.2.1.51, NEB), 400 U/mL; almond meal α-fucosidase (AMF, releases nonreducing terminal fucose residues linked via α(1–3) and α(1–4), but not core α(1–6) fucose, EC 3.2.1.111, NEB), 40 U/mL and β-*N*-acetylglucosaminidase cloned from *S. pneumoniae*, expressed in *Escherichia coli* (GUH, digests β-linked *N*- acetylglucosamine (GlcNAc) residues, but not bisecting GlcNAc β(1–4) linked to mannose, EC 3.2.1.30, Agilent), 8 U/mL. After incubation, enzymes were removed by filtration through a 10 kDa protein-binding EZ filters (Millipore Corporation)^31^. Following digestion, the enzymes were removed by microcentrifuge filtration and the oligosaccharides were analysed by HILIC-UPLC.

### Derived features calculations

Groups of GPs were defined from their common features, as follows^12^:

Oligomannose (OligoM): (GP6/2) + GP11; Fucosylation: Core-fucose (Core F) (GP2 + GP5 + (GP6/2) + GP8 − 10 + GP14 − 15 + GP17 − 18 + GP22 − 23 + GP27 − 28 + GP36 + (GP44/2)) and outer-arm fucose (Outer arm F) (GP37 + GP40 + (GP41/3) + GP45 + (GP46/3)); Branching: A1 (monoantennary, GP1 − 3 +(GP12/2) + (GP21/2)); A2 (biantennary, GP4 − 5 + (GP6/2) + GP7 − 10 + (GP12/2) + GP13 − 20 + (GP21/2) + GP22 − 28); A3 (triantennary, GP29 + GP31−37); A4 (tetraantennary, GP30 + GP38−46); Galactosylation: G0 (agalactosylated, GP1 − 2 + GP4 − 5 + (GP6/2) + (GP12/2)); G1 (monogalactosylated, GP3 + GP7−10 + (GP12/2) + GP16 − 18 + (GP21/2)); G2 (digalactosylated, GP13 − 15 + GP19 + GP20 + (GP21/2) + GP22 − 28); G3 (trigalactosylated, GP29 + GP31−37); G4 (tetragalactosylated, GP30 + GP38−46); Sialylation: S0 (neutral (asialylated), GP1 − 15); S1 (monosialylated, GP16 − 23 + GP30); S2 (disialylated, GP24 − 29 + GP31); S3 (trisialylated, GP32 − 40); S4 (tetrasialylated, GP41 − 46).

### Data analyses and statistics

Average values from duplicates were taken for comparison between ISF and plasma group (Table S2). Statistical significance was performed using Paired T test (most GPs and features were normally distributed (Shapiro Wilk test)) using IBM SPSS Statistics Version 29.0.1.0 (171). Adjustment for multiple testing was performed by Benjamini Hochberg (BH) method using false discovery rate 0.05. For the PCA, the data were firstly scaled using fit_transform function and PCA plots were done using Python 3.8.8 associated Matplotlib package. Plots of average % peak areas were performed using GraphPad Prism (version 10.1.0).

## Supplementary Information

Below is the link to the electronic supplementary material.

Supplementary material 1

Supplementary material 2

## Data Availability

Raw MS data for structural interpretation of *N*-glycans from ISF and plasma pooled samples are available on GlycoPOST (https://glycopost.glycosmos.org/) using the Project ID no. GPST000651. Python Jupyter notebooks and associated datasets are available at [https://github.com/RadkaFahey/ISF_paper](https:/github.com/RadkaFahey/ISF_paper).

## References

[1] Ghazarian, H., Idoni, B. & Oppenheimer, S. B. A glycobiology review: Carbohydrates, lectins and implications in cancer therapeutics. *Acta Histochem.* **113**, 236–247. 10.1016/j.acthis.2010.02.004 (2011). 20199800 10.1016/j.acthis.2010.02.004 PMC3027850

[2] 2 Marino, K., Saldova, R., Adamczyk, B. & Rudd, P. M. in *Carbohydrate Chemistry: Chemical and Biological Approaches* Vol. 37 (ed Amelia Pilar Rauter)RSC Publishing (2012).

[3] Samant, P. P. et al. Sampling interstitial fluid from human skin using a microneedle patch. *Sci. Transl. Med.*10.1126/scitranslmed.aaw0285 (2020). 33239384 10.1126/scitranslmed.aaw0285 PMC7871333

[4] Friedel, M. et al. Opportunities and challenges in the diagnostic utility of dermal interstitial fluid. *Nat. Biomed. Eng.* **7**, 1541–1555. 10.1038/s41551-022-00998-9 (2023). 36658344 10.1038/s41551-022-00998-9

[5] Heikenfeld, J. et al. Accessing analytes in biofluids for peripheral biochemical monitoring. *Nat. Biotechnol.* **37**, 407–419. 10.1038/s41587-019-0040-3 (2019). 30804536 10.1038/s41587-019-0040-3

[6] Oharazawa, A., Maimaituxun, G., Watanabe, K., Nishiyasu, T. & Fujii, N. Metabolome analyses of skin dialysate: Insights into skin interstitial fluid biomarkers. *J. Dermatol. Sci.* **114**, 141–147. 10.1016/j.jdermsci.2024.04.001 (2024). 38740531 10.1016/j.jdermsci.2024.04.001

[7] Tran, B. Q. et al. Proteomic Characterization of Dermal Interstitial Fluid Extracted Using a Novel Microneedle-Assisted Technique. *J. Proteome Res.* **17**, 479–485. 10.1021/acs.jproteome.7b00642 (2018). 29172549 10.1021/acs.jproteome.7b00642

[8] Upton, T. J. et al. High-resolution daily profiles of tissue adrenal steroids by portable automated collection. *Sci. Transl. Med.* **15**, eadg8464. 10.1126/scitranslmed.adg8464 (2023). 37343084 10.1126/scitranslmed.adg8464

[9] Chatard, C., Meiller, A. & Marinesco, S. Microelectrode biosensors for in vivo analysis of brain interstitial fluid. *Electroanalysis* **30**, 977–998 (2018).

[10] Terkelsen, T. et al. N-glycan signatures identified in tumor interstitial fluid and serum of breast cancer patients: Association with tumor biology and clinical outcome. *Mol. Oncol.* **12**, 972–990. 10.1002/1878-0261.12312 (2018). 29698574 10.1002/1878-0261.12312 PMC5983225

[11] Wang, Z. et al. Microneedle patch for the ultrasensitive quantification of protein biomarkers in interstitial fluid. *Nat Biomed Eng* **5**, 64–76. 10.1038/s41551-020-00672-y (2021). 33483710 10.1038/s41551-020-00672-y PMC8020465

[12] Saldova, R. et al. Association of N-glycosylation with breast carcinoma and systemic features using high-resolution quantitative UPLC. *J Proteome Res* **13**, 2314–2327. 10.1021/pr401092y (2014). 24669823 10.1021/pr401092y

[13] Stumpo, K. A. & Reinhold, V. N. The N-glycome of human plasma. *J Proteome Res* **9**, 4823–4830. 10.1021/pr100528k (2010). 20690605 10.1021/pr100528k PMC2933516

[14] Dotz, V. & Wuhrer, M. N-glycome signatures in human plasma: Associations with physiology and major diseases. *FEBS Lett* **593**, 2966–2976. 10.1002/1873-3468.13598 (2019). 31509238 10.1002/1873-3468.13598

[15] Pongracz, T., Mayboroda, O. A. & Wuhrer, M. The human blood N-Glycome: Unraveling disease glycosylation patterns. *JACS Au* **4**, 1696–1708. 10.1021/jacsau.4c00043 (2024). 38818049 10.1021/jacsau.4c00043 PMC11134357

[16] Maslov, D. E. et al. Fast and simple protocol for N-Glycome analysis of human blood plasma proteome. *Biomolecules*10.3390/biom14121551 (2024). 39766258 10.3390/biom14121551 PMC11673551

[17] Adamczyk, B., Struwe, W. B., Ercan, A., Nigrovic, P. A. & Rudd, P. M. Characterization of fibrinogenglycosylation and its importance for serum/plasma N-glycome analysis. *J Proteome Res.* **12**, 444-454 10.1021/pr300813h (2013). 10.1021/pr300813h 23151259

[18] Kattla, J. J. et al. Elsevier,. in *Comprehensive Biotechnology* Vol. 3 (ed Murray Moo-Young) 467–486 (2011).

[19] Wiig, H. & Swartz, M. A. Interstitial fluid and lymph formation and transport: Physiological regulation and roles in inflammation and cancer. *Physiol. Rev.* **92**, 1005–1060. 10.1152/physrev.00037.2011 (2012). 22811424 10.1152/physrev.00037.2011

[20] Aukland, K. & Reed, R. K. Interstitial-lymphatic mechanisms in the control of extracellular fluid volume. *Physiol. Rev.* **73**, 1–78. 10.1152/physrev.1993.73.1.1 (1993). 8419962 10.1152/physrev.1993.73.1.1

[21] Miller, P. R. et al. Extraction and biomolecular analysis of dermal interstitial fluid collected with hollow microneedles. *Commun. Biol.* **1**, 173. 10.1038/s42003-018-0170-z (2018). 30374463 10.1038/s42003-018-0170-z PMC6197253

[22] Royle, L. et al. HPLC-based analysis of serum N-glycans on a 96-well plate platform with dedicated database software. *Anal. Biochem.* **376**, 1–12. 10.1016/j.ab.2007.12.012 (2008). 18194658 10.1016/j.ab.2007.12.012

[23] Lauc, G. et al. Genomics meets glycomics—The first GWAS study of human N-glycome identifies HNF1α as a master regulator of plasma protein fucosylation. *PLoS Genet.* **6**, e1001256. 10.1371/journal.pgen.1001256 (2010). 21203500 10.1371/journal.pgen.1001256 PMC3009678

[24] Knezevic, A. et al. Variability, heritability and environmental determinants of human plasma N-glycome. *J. Proteome Res.* **8**, 694–701. 10.1021/pr800737u (2009). 19035662 10.1021/pr800737u

[25] Barbone, A. S., Meftah, M., Markiewicz, K. & Dellimore, K. Beyond wearables and implantables: A scoping review of insertable medical devices. *Biomed. Phys. Eng. Express* **5**, 062002. 10.1088/2057-1976/ab4b32 (2019).

[26] Tehrani, F. et al. An integrated wearable microneedle array for the continuous monitoring of multiple biomarkers in interstitial fluid. *Nat. Biomed. Eng.* **6**, 1214–1224. 10.1038/s41551-022-00887-1 (2022). 35534575 10.1038/s41551-022-00887-1

[27] Kim, Y. & Prausnitz, M. R. Sensitive sensing of biomarkers in interstitial fluid. *Nat. Biomed. Eng.* **5**, 3–5. 10.1038/s41551-020-00679-5 (2021). 33483708 10.1038/s41551-020-00679-5

[28] Himawan, A. et al. Where microneedle meets biomarkers: Futuristic application for diagnosing and monitoring localized external organ diseases. *Adv. Healthc. Mater.* **12**, e2202066. 10.1002/adhm.202202066 (2023). 36414019 10.1002/adhm.202202066 PMC11468661

[29] Chachaj, A., Matkowski, R., Grobner, G., Szuba, A. & Dudka, I. Metabolomics of interstitial fluid, plasma and urine in patients with arterial hypertension: New insights into the underlying mechanisms. *Diagnostics (Basel)*10.3390/diagnostics10110936 (2020). 33187152 10.3390/diagnostics10110936 PMC7698256

[30] Wu, Z. et al. Interstitial fluid-based wearable biosensors for minimally invasive healthcare and biomedical applications. *Commun. Mater.* **5**, 33. 10.1038/s43246-024-00468-6 (2024).

[31] Royle, L., Radcliffe, C. M., Dwek, R. A. & Rudd, P. M. Detailed structural analysis of N-glycans released from glycoproteins in SDS-PAGE gel bands using HPLC combined with exoglycosidase array digestions. *Methods Mol. Biol.* **347**, 125–143. 10.1385/1-59745-167-3:125 (2006). 17072008 10.1385/1-59745-167-3:125

[32] Kuster, B., Wheeler, S. F., Hunter, A. P., Dwek, R. A. & Harvey, D. J. Sequencing of N-linked oligosaccharides directly from protein gels: In-gel deglycosylation followed by matrix-assisted laser desorption/ionization mass spectrometry and normal-phase high-performance liquid chromatography. *Anal. Biochem.* **250**, 82–101. 10.1006/abio.1997.2199 (1997). 9234902 10.1006/abio.1997.2199

[33] Bigge, J. C. et al. Nonselective and efficient fluorescent labeling of glycans using 2-amino benzamide and anthranilic acid. *Anal. Biochem.* **230**, 229–238. 10.1006/abio.1995.1468 (1995). 7503412 10.1006/abio.1995.1468

[34] Watanabe, Y., Aoki-Kinoshita, K. F., Ishihama, Y. & Okuda, S. GlycoPOST realizes FAIR principles for glycomics mass spectrometry data. *Nucleic Acids Res.* **49**, D1523–D1528. 10.1093/nar/gkaa1012 (2021). 33174597 10.1093/nar/gkaa1012 PMC7778884

