# Supplementary material for: Interstitial fluid N-glycans serve as a proxy for serum biomarker discovery in a pilot study
Source: Sci Rep. 2026 Jul 17;16:22500. doi: 10.1038/s41598-026-51768-5 (PMC13376865; doi:10.1038/s41598-026-51768-5)
Supplement: Supplementary file 2 — Supplementary material 2 [file 41598_2026_51768_MOESM2_ESM.docx]

**ASSOCIATED CONTENT.**

**Supplementary Tables.**

**Table S1.** Mass spectrometry data for ISF and plasma pool from the same individuals.

**Table S2.** Average and individual peak % areas for each subject

**Table S3**. Statistical evaluation of ISF and plasma GPs and glycan features
